# Omega-3 Fatty Acids and Markers of Thrombosis in Patients with Atrial Fibrillation

**DOI:** 10.3390/nu16020178

**Published:** 2024-01-05

**Authors:** Martin F. Reiner, Daniela A. Bertschi, Laura Werlen, Andrea Wiencierz, Stefanie Aeschbacher, Pratintip Lee, Nicolas Rodondi, Elisavet Moutzouri, Leo Bonati, Tobias Reichlin, Giorgio Moschovitis, Jonas Rutishauser, Michael Kühne, Stefan Osswald, David Conen, Jürg H. Beer

**Affiliations:** 1 Department of Cardiology, University Heart Center Zurich, 8091 Zurich, Switzerland; martin.reiner@gmx.at; 2 Department of Internal Medicine, Cantonal Hospital of Baden, 5404 Baden, Switzerland; 3 Department of Clinical Research, University of Basel, University Hospital Basel, 3010 Basel, Switzerland; 4 Department of Cardiology, University Hospital Basel, 4056 Basel, Switzerland; 5 Cardiovascular Research Institute Basel, University Hospital Basel, 4056 Basel, Switzerland; 6 Center for Molecular Cardiology, Laboratory for Platelet Research, University of Zurich, 8952 Zurich, Switzerland; 7 Department of General Internal Medicine, Inselspital, Bern University Hospital, 3010 Bern, Switzerland; 8 Institute of Primary Health Care (BIHAM), University of Bern, 3010 Bern, Switzerland; 9 Department of Neurology and Stroke Center, University Hospital Basel, 4031 Basel, Switzerland; 10 Department of Cardiology, Inselspital, Bern University Hospital, 3010 Bern, Switzerland; 11 Division of Cardiology, Ospedale Regionale di Lugano, 6900 Ticino, Switzerland; 12 Population Health Research Institute, McMaster University, Hamilton, ON L8L 2X2, Canada

**Keywords:** atrial fibrillation, beta-thromboglobulin, D-dimer, omega-3 fatty acids, stroke

## Abstract

Omega-3 fatty acids (*n*-3 FAs) are associated with a lower risk of ischemic stroke in patients with atrial fibrillation (AF). Antithrombotic mechanisms may in part explain this observation. Therefore, we examined the association of *n*-3 FAs with D-dimer and beta-thromboglobulin (BTG), markers for activated coagulation and platelets, respectively. The *n*-3 FAs eicosapentaenoic acid (EPA), docosahexaenoic acid (DHA), docosapentaenoic acid (DPA) and alpha-linolenic acid (ALA) were determined via gas chromatography in the whole blood of 2373 patients with AF from the Swiss Atrial Fibrillation cohort study (ClinicalTrials.gov Identifier: NCT02105844). In a cross-sectional analysis, we examined the association of total *n*-3 FAs (EPA + DHA + DPA + ALA) and the association of individual fatty acids with D-dimer in patients with detectable D-dimer values (*n* = 1096) as well as with BTG (*n* = 2371) using multiple linear regression models adjusted for confounders. Median D-dimer and BTG levels were 0.340 ug/mL and 448 ng/mL, respectively. Higher total *n*-3 FAs correlated with lower D-dimer levels (coefficient 0.94, 95% confidence interval (Cl) 0.90–0.98, *p* = 0.004) and lower BTG levels (coefficient 0.97, Cl 0.95–0.99, *p* = 0.003). Likewise, the individual *n*-3 FAs EPA, DHA, DPA and ALA showed an inverse association with D-dimer. Higher levels of DHA, DPA and ALA correlated with lower BTG levels, whereas EPA showed a positive association with BTG. In patients with AF, higher levels of *n*-3 FAs were associated with lower levels of D-dimer and BTG, markers for activated coagulation and platelets, respectively. These findings suggest that *n-3* FAs may exert antithrombotic properties in patients with AF.

## 1. Introduction

Atrial fibrillation (AF) is the most common sustained cardiac arrhythmia affecting over 2% of the adult population, and cardioembolic stroke is a major complication of AF causing substantial morbidity and mortality [1,2]. Oral anticoagulation reduces the risk of thromboembolic complications; however, a considerable risk remains [3,4], and additional strategies are needed to reduce the burden of AF. The randomized controlled REDUCE-IT trial found that the Omega-3 fatty acid (*n*-3 FA) eicosapentaenoic acid (EPA) reduces the risk of stroke in patients with cardiovascular disease [5] and cohort studies reported an inverse correlation of *n*-3 FAs with ischemic stroke in patients with AF [6,7]. The antithrombotic properties of *n*-3 FAs may partly explain these observations. Therefore, we investigate the association of *n*-3 FAs with D-dimer and beta-thromboglobulin (BTG), biomarkers of coagulation and platelet activation, respectively, that play a pivotal role in AF [8].

D-dimer is generated after plasmin-mediated degradation of cross-linked fibrin polymers [9]. It serves as an established marker for activated coagulation and fibrinolysis [9] and is widely used in the diagnostic work-up for deep vein thrombosis and pulmonary embolism, among others [10]. Levels of D-dimer are increased in patients with AF compared to patients in sinus rhythm [11], and D-dimer levels predict cardioembolic stroke in AF patients [12,13,14]. BTG is stored in alpha-granules of platelets and is released upon stimulation by various platelet agonists [15]. Similar to D-dimer, levels of BTG are higher in patients with AF compared with patients in sinus rhythm and are suppressed by oral anticoagulation [11]. Furthermore, BTG levels are associated with the prevalence of thrombi in left atrial appendages of AF patients [16]. 

In this large cross-sectional analysis of the Swiss Atrial Fibrillation (Swiss-AF) cohort study of patients with AF, we investigate the association of total *n*-3 FAs and the associations of the individual *n*-3 FAs—EPA, docosahexaenoic acid (DHA), docosapentaenoic acid (DPA) and alpha-linolenic acid (ALA)—with D-dimer and BTG.

## 2. Methods

### 2.1. Study Population

The current study is an analysis within the Swiss-AF cohort study (ClinicalTrials.gov Identifier: NCT02105844), a national prospective multicenter study of 2415 patients recruited from 14 centers in Switzerland with the aim of investigating a potential association of brain damage and cognitive decline in patients suffering from AF [17]. Patients aged ≥65 years and with either paroxysmal, persistent or permanent AF were included. Exclusion criteria were short episodes of reversible forms of AF (e.g., after surgery or severe sepsis), inability to give informed consent and acute illness within the last four weeks [17]. Paroxysmal AF was defined as lasting ≤ 7 days without requiring cardioversion. Persistent AF was defined as AF lasting more than 7 days or requiring intervention. In the case of unsuccessful cardioversion or no rhythm control intervention, AF was categorized as permanent AF [17]. 

**Figure 1:**
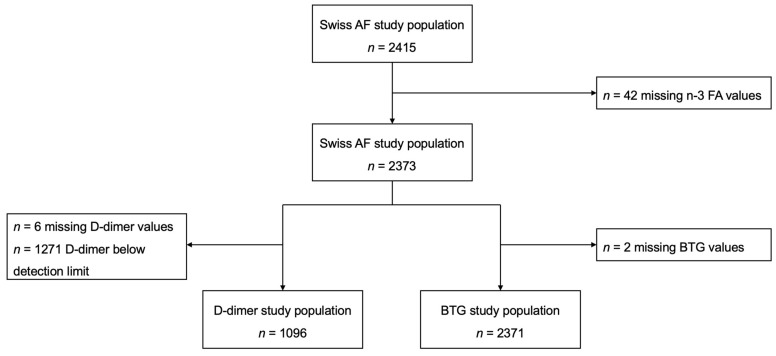
Flow chart of study population. BTG = beta-thromboglobulin, *n*-3 FA = Omega-3 fatty acid, Swiss AF = Swiss atrial fibrillation.

Of the total 2415 patients included in the Swiss-AF cohort study, 2373 were included in the current study (42 patients excluded due to missing values for *n*-3 FA). For the D-dimer, 1096 patients were included (1271 with a D-dimer below the detection limit and 6 patients with missing values for D-dimer were excluded), and for BTG, 2371 patients were included (2 patients with missing values for BTG were excluded) in this study (Figure 1).

### 2.2. Quantification of Whole Blood n-3 FAs

For every patient, non-fasting venous EDTA-anticoagulated blood was collected at the baseline visit according to standard procedures. Samples of 1 mL were aliquoted immediately and stored at −80 °C in a centralized biobank at the University Hospital Basel [17]. Samples were transported to Germany on dry ice without additional freeze–thaw cycles. Whole-blood fatty acids were analyzed at Omegametrix GmbH, Martinsried, Germany, using the HS-Omega-3 Index^®^ methodology for analyzing whole-blood fatty acid compositions as described previously. [18,19] Fatty acid methyl esters were made from red blood cells by acid transesterification. Afterward, they were analyzed using gas chromatography via a GC2010 Gas Chromatograph (Shimadzu, Duisburg, Germany) equipped with a 100 m SP2560 column (Supelco, Bellefonte, PA, USA) using hydrogen as the carrier gas. Specific fatty acids were identified by comparing them with a mixture of fatty acids typical for red blood cells. The results are provided as percentages of fatty acids (EPA, DHA, DPA, ALA or total *n*-3 FAs [EPA + DHA + DPA + ALA]) after response factor correction. The coefficient for variation was 5%. Analyses were quality-controlled according to DIN ISO 15189.

### 2.3. Quantification of BTG and D-Dimer

At study baseline, non-fasting venous lithium heparin plasma samples were drawn according to standard operating procedures. The samples were processed immediately by centrifugation at 3000 rounds per minute for 7 min, aliquoted into 1 mL cryotubes and stored at −80 °C in a centralized biobank at the University Hospital Basel. BTG levels were measured using a multiplexed particle-based flow cytometric cytokine assay [20]. A BTG-Luminex kit was purchased from Biotechne (Oxon, UK). The procedures were caried out according to the manufacturer’s instructions using a conventional flow cytometer (Guava EasyCyte 8HT, Millipore, Zug, Switzerland). Values are provided in ng/mL.

D-dimer values were analyzed via a Tina-quant D-Dimer Gen.2 (Roche, Basel, Switzerland) using a cobas C311 analyzer (Roche) according to the manufacturer’s instructions. Values are provided in ug/mL. The minimal determined D-dimer value was 0.150 ug/mL.

### 2.4. Statistical Analysis

We used multiple linear regression models to assess the association between total *n*-3 FAs and the individual *n*-3 FAs (ALA, DHA, DPA or EPA) with D-dimer and BTG. Due to their right-skewed distributions, we used the natural logarithm of these biomarkers as the outcomes. We transformed the estimated coefficients back to the original scales of D-dimer and BTG. The reported coefficients represent multiplicative effects on the geometric means of the distributions of D-dimer and BTG, respectively. For the analyses of D-dimer and BTG, we present three models for *n*-3 FA total as a predictor and all component parts as a predictor each, for a total of 6 models (3 for D-dimer and 3 for BTG). Model 1 was adjusted for age at baseline and sex. Model 2 was additionally adjusted for body mass index, physical activity status, smoking and drinking habits, type of AF, hypertension, diabetes mellitus, chronic kidney disease, cancer, previous stroke, previous transient ischemic attack, heart failure, coronary artery disease, major bleeding, aspirin use, statin use, P2Y12 inhibitor use and use of/type of anticoagulant. Finally, due to the well-described relationship of inflammation and thrombogenesis, [21,22] model 3 was adjusted for high-sensitivity C-reactive protein. All analyses were performed in R version 4.2.2 Patched (2022-11-10 r83330) or higher (R Core Team, 2022). The study was performed according to STROBE guidelines for cross-sectional studies. 

## 3. Results

### 3.1. Study Population

The mean age of patients included in the analysis for D-dimer and BTG was 75 years and 73 years, respectively, and 27% were female. Paroxysmal AF was the most common type of AF. The vast majority of patients were anticoagulated and more than half of the patients were treated with new oral anticoagulants (Table 1 and Table 2). The mean total *n*-3 FAs, consisting of EPA, DHA, DPA and ALA, was 6.0% (standard deviation (SD) 1.2%). Mean (SD) values for the individual components are depicted in Table 3. 

### 3.2. Association of Omega-3 Fatty Acids and D-Dimer

The median D-dimer in patients with detectable D-dimer values was 0.340 ug/mL. We found an inverse association of total *n*-3 FAs with D-dimer (coefficient 0.94, 95% confidence interval (Cl) 0.90–0.98, *p* = 0.004) after adjustment for multiple confounders (Table 4). Thus, a one percentage point increase in total *n*-3 FAs was associated with a 6% lower D-dimer value. Similarly, the individual *n*-3 FA EPA (coefficient 0.91, 95% Cl 0.76–1.10, *p* = 0.336), DHA (coefficient 0.95, 95% Cl 0.89–1.01, *p* = 0.124), DPA (coefficient 0.93, 95% Cl 0.78–1.12, *p* = 0.452) and ALA (coefficient 0.95, 95% Cl 0.63–1.45, *p* = 0.827) showed an inverse association with D-dimer (Table 4).

### 3.3. Association of Omega-3 Fatty Acids and Beta-Thromboglobulin

The median BTG was 448 ng/mL. Total *n*-3 FAs were inversely associated with beta-thromboglobulin, a marker of platelet activation (coefficient 0.97, Cl 0.95–0.99, *p* = 0.003) after adjustment for multiple confounders (Table 5). Thus, a one percentage point increase in total *n*-3 FAs was associated with a 3% lower BTG value. Likewise, DHA (coefficient 0.94, Cl 0.92–0.97, *p* < 0.001), DPA (coefficient 0.91, Cl 0.84–0.98, *p* = 0.019) and ALA (coefficient 0.83, Cl 0.69–1.01, *p* = 0.060) showed an inverse association with BTG, whereas EPA showed a positive association with BTG (coefficient 1.12, Cl 1.03–1.22, *p* = 0.010) after adjustment for multiple confounders (Table 5).

## 4. Discussion

In this large cross-sectional study of the Swiss-AF cohort study of patients with AF, we investigated the association of total *n*-3 FAs and its individual components with D-dimer and BTG, markers of coagulation and platelet activation, respectively. We found an inverse association of total *n*-3 FAs with both D-dimer and BTG after adjustment for multiple confounders, including high-sensitivity C-reactive protein. These observations suggest potential antithrombotic properties of *n*-3 FAs in patients with AF and may in part explain the lower risk of ischemic stroke observed in patients with AF and high blood levels of *n*-3 FAs [6,7]. Similar to total *n*-3 FAs, the individual components of EPA, DHA, DPA and ALA were inversely associated with D-dimer. Finally, the *n*-3 FAs DHA, DPA and ALA showed an inverse association with BTG, whereas EPA was associated with higher BTG values.

The inverse associations of *n*-3 FAs with D-dimer and BTG are in line with our hypothesis, while the positive association of EPA with BTG was unexpected and appears contradictory to earlier reports. Platelet activation results in the release of alpha-granules, which in turn increases plasma levels of BTG [15]. Previous studies found that EPA prevents platelet activation [23,24], and in line with this, EPA administration reduces BTG levels in humans after 2 weeks of supplementation [25]. On the other hand, the administration of fish oil including a mixture of *n*-3 FAs did not affect BTG levels in non-insulin-dependent diabetic patients [26], and high plasma EPA levels were not associated with BTG in patients with or without peripheral artery disease [27]. These studies suggest that supplementation of purified EPA increases BTG, but supplementation with a mixture of *n*-3 FAs does not affect BTG levels, possibly due to opposing effects of different *n*-3 FAs. Furthermore, the effect may be dependent on EPA supplementation rather than on nutritionally achievable EPA levels, and it cannot be ruled out that the effect of EPA on BTG levels was a short-term observation, since the study by Yoshimura et al. investigated BTG levels 2 weeks following EPA administration. [25] Lastly, the inverse relation of EPA and BTG observed in our study may not be explained by higher platelet activation but rather by a higher platelet count, since EPA, but not DHA, was shown to increase platelet numbers [28].

DHA and DPA reduce platelet aggregation in response to various agonists [23,24], and ALA reduces murine platelet aggregation as well as glycoprotein Ib clustering and subsequent binding to von Willebrand factor in human platelets [29]. The latter findings support our observations. The inverse association of *n*-3 FAs with D-dimer is also in line with our hypothesis and other reports describing anticoagulant effects of EPA, DHA and ALA in experimental [29,30] as well as observational studies. [18,31]

In our previous analysis of the Swiss AF study, we showed that higher levels of EPA were associated with a lower prevalence of ischemic brain infarcts [6]. The inverse association of EPA with lower D-dimer in the current study is in line with this finding and may represent a mechanistic link. Although we also found that total *n*-3 FAs were associated with lower levels of D-dimer in the current study, total *n*-3 FAs did not correlate with the prevalence of ischemic brain lesions in AF patients [6], which supports the concept of distinct properties of individual *n*-3 FAs and suggests that additional mechanisms play a role in the pathogenesis of ischemic stroke in AF patients.

Interestingly, all individual *n*-3 FAs showed an inverse correlation with D-dimer, but only the relationship of total *n*-3 FAs with D-dimer reached statistical significance. A possible explanation could be that the individual fatty acids exhibit a weak association, which becomes significant only when analyzed combined. This finding may be due to the lower number of patients included in the D-dimer analysis. With the exception of EPA, the inverse correlations of *n*-3 FAs with BTG were stronger and reached statistical significance, as was the case for the individual *n*-3 FAs DHA and DPA, which is probably due to the higher number of patients in the BTG analysis.

Previous studies on plant-derived *n*-3 FAs found that dietary intake of ALA was not associated with ischemic stroke in middle-aged Danish men and women; likewise, the authors found no association with cardioembolic stroke in this cohort, whereas it should be noted that less than 1% of the cohort reported having AF or atrial flutter [32]. Similarly, the adipose tissue content of ALA was not significantly associated with total ischemic strokes or cardioembolic strokes [33]. These findings are in line with our previous observation that ALA, measured in whole blood, has no significant association with small or large ischemic brain lesions, determined by brain magnetic resonance imaging in patients with AF [6]. They are also in line with the current study, which found no significant associations of ALA with D-dimer or BTG.

A meta-analysis of randomized controlled trials found that supplementation of *n*-3 FAs increases the risk of AF in a dose-dependent manner, whereas patients supplemented with high dosages of *n*-3 FAs (>1 g/day) experienced a higher risk of developing AF [34]. On the contrary, a recent meta-analysis of observational studies reported that the risk of AF was not increased in patients with habitual intake of *n*-3 FAs [35]. The latter finding is in line with a previous analysis of the Swiss AF study, showing no significant association of total *n*-3 FAs with the type of AF. In patients with known AF, neither total nor individual fish- or plant-derived *n*-3 FAs showed a significant correlation with an increased AF burden, i.e., non-paroxysmal AF [36]. Whether *n*-3 FA supplementation in low or high dosages affects the burden of AF in patients with known AF remains to be determined in future randomized controlled trials. Furthermore, the AF burden is unlikely to contribute to the associations between *n*-3 FAs and thrombotic markers in this observational study.

Antithrombotic properties of *n*-3 FAs may be associated with a higher bleeding risk, particularly in the presence of anticoagulation, as is the case in most patients with AF. A recent meta-analysis of randomized controlled trials in patients at cardiovascular risk or with established cardiovascular disease reported higher bleeding events in patients supplemented with high (>1 g per day), but not with low dosages of *n*-3 FAs (<1 g per day) [37]. On the other hand, a sub-analysis of the REDUCE-IT trial found no significant association of high-dosage EPA (4 g/day) with bleeding events in patients with or without AF [38]. Similarly, our previous analysis found no correlation of *n*-3 FAs with cerebral microbleeds in AF patients largely treated with oral anticoagulation [6], suggesting that nutritionally achieved *n*-3 FA levels may not affect bleeding risk.

D-dimer is a strong and independent risk predictor for stroke in patients with AF [14,39], highlighting the relevance of our findings. Notably, this association was observed in patients largely treated with oral anticoagulation, known to reduce D-dimer [12], which suggests a potential benefit of *n*-3 FAs on top of oral anticoagulation.

The strengths of our study include the large and well-characterized population of patients with AF. Further, we determined *n*-3 FAs in whole blood, which represents the average long-term fatty acid intake (including potential *n*-3 FA supplementation) and is independent of individual bioavailability; furthermore, it is considered to be stable over a long time. [40] Finally, we observed an inverse association of *n*-3 FAs with D-dimer despite the fact that the majority of patients were on oral anticoagulation known to reduce D-dimer levels [12]. The limitations of our study are the exclusion of patients with undetectable D-Dimer values, which could have introduced a selection bias. Although patients with acute illness within 4 weeks before enrolment were excluded from the study and median high-sensitivity C-reactive protein levels were low in the D-dimer population (2.4 mg/L), the lack of information on anti-inflammatory and anti-infective drugs, which could have affected D-dimer levels, is a potential missing confounder that was not considered in this analysis. The study was not corrected for platelet counts and international normalized ratios due to the lack of data. The cross-sectional study design does not allow drawing causal relationships or to assess the directionality of the observed associations. The study was performed in Switzerland in patients ≥65 years of age, and therefore, the results may not apply to other geographic areas and younger people. Despite extensive adjustments, there is a residual risk of confoundment. Future studies should therefore include a wider range and more diverse population to enhance generalizability, and furthermore, longitudinal studies are needed to establish causality.

The findings of our study suggest that *n*-3 FAs may have antithrombotic properties in patients with AF who are on oral anticoagulation. These results provide a potential pathophysiological explanation of our previous findings, which showed that *n*-3 FAs, particularly EPA, are inversely correlated with ischemic brain infarcts in patients with AF [6]. Although the anticoagulatory effect appears to be paramount for stroke prevention in AF [41], an additional antiaggregatory effect is likely to contribute to stroke prevention [42]. The fact that the AF burden increases with *n*-3 FA treatment [34] may be of less relevance in patients who already have AF. Additional longitudinal studies are required to confirm the association between blood *n*-3 FAs and cardioembolic stroke in AF patients, and future studies should also address bleeding complications as well as the underlying mechanisms. Despite the increase in AF burden upon high dosages of *n*-3 FA supplementation [34], the recent REDUCE-IT study found a marked reduction in strokes – the major complication of AF – in patients with cardiovascular disease [38]. A randomized controlled trial examining the effect of *n*-3 FA supplementation on top of oral anticoagulation in patients with atrial fibrillation is warranted.

## 5. Conclusions

In conclusion, this cross-sectional study of AF patients showed an inverse association of *n*-3 FAs with D-dimer and BTG, markers of activated coagulation and platelets, respectively. These findings suggest the antithrombotic properties of *n*-3 FAs in patients with AF treated with oral anticoagulation.

## Figures and Tables

**Table 1:** Baseline characteristics of D-dimer study population. IQR = interquartile range, SD = standard deviation.

Baseline Characteristics of D-Dimer Study Population, *n* = 1096	
Mean age (SD)	74.9 (8.4)
Female (%)	291 (26.6)
Median body mass index (IQR)	27.2 (24.4, 30.8)
Physical activity (%)	424 (38.7)
Smoker (%)	
Never	474 (43.2)
Past	535 (48.8)
Active	85 (7.8)
Median alcohol units per day (IQR)	0.4 (0.1, 1.2)
AF type (%)	
Paroxysmal	483 (44.1)
Persistent	310 (28.3)
Permanent	303 (27.6)
Hypertension (%)	805 (73.4)
Diabetes mellitus (%)	238 (21.7)
Chronic kidney disease (%)	315 (28.7)
Cancer (%)	192 (17.5)
Stroke (%)	155 (14.1)
Transient ischemic attack (%)	107 (9.8)
Heart failure (%)	355 (32.4)
Coronary artery disease (%)	399 (36.4)
Major bleeding (%)	99 (9.0)
Aspirin (%)	262 (23.9)
Statin (%)	557 (50.8)
P2Y12 inhibitor (%)	79 (7.2)
Type of anticoagulation	
Novel oral anticoagulants (%)	499 (45.5)
Vitamin K antagonist (%)	417 (38.0)
None (%)	180 (16.4)

**Table 2:** Baseline characteristics of beta-thromboglobulin study population. IQR = interquartile range, SD = standard deviation.

Baseline Characteristics Beta-Thromboglobulin Study Population, *n* = 2371	
Mean age (SD)	73.2 (8.4)
Female (%)	644 (27.2)
Median body mass index (IQR)	27.0 (24.4, 30.3)
Physical activity (%)	1093 (46.1)
Smoker (%)	
Never	1038 (43.8)
Past	1158 (48.8)
Active	173 (7.3)
Median alcohol units per day (IQR)	0.5 (0.1, 1.3)
AF type (%)	
Paroxysmal	1058 (44.6)
Persistent	724 (30.5)
Permanent	589 (24.8)
Hypertension (%)	1653 (69.7)
Diabetes mellitus (%)	413 (17.4)
Chronic kidney disease (%)	502 (21.2)
Cancer (%)	383 (16.2)
Stroke (%)	312 (13.2)
Transient ischemic attack (%)	213 (9.0)
Heart failure (%)	618 (26.1)
Coronary artery disease (%)	716 (30.2)
Major bleeding (%)	148 (6.2)
Aspirin (%)	395 (16.7)
Statin (%)	1169 (49.3)
P2Y12 inhibitor (%)	146 (6.2)
Type of anticoagulation	
Novel oral anticoagulants (%)	1209 (51.0)
Vitamin K antagonist (%)	934 (39.4)
None (%)	228 (9.6)

**Table 3:** Omega-3 fatty acid fractions. Whole-blood total and individual *n*-3 FAs are expressed as percentages of total identified fatty acids. Total omega-3 fatty acids = EPA + DHA + DPA + ALA. ALA = alpha-linolenic acid, DHA = docosahexaenoic acid, DPA = docosapentaenoic acid, EPA = eicosapentaenoic acid, SD = standard deviation, *n*-3 FAs = Omega-3 fatty acids.

Overall Population, *n* = 2373	Mean Fatty Acid Fraction, % (SD)
Total Omega-3 fatty acids	6.0 (1.2)
Eicosapentaenoic acid (EPA)	0.8 (0.3)
Docosahexaenoic acid (DHA)	3.3 (0.8)
Docosapentaenoic acid (DPA)	1.7 (0.3)
Alpha-linolenic acid (ALA)	0.2 (0.1)

**Table 4:** Associations of *n*-3 FAs with D-dimer. Model 1 was adjusted for age and sex. Model 2 was additionally adjusted for body mass index, physical activity, smoking, alcohol units per day, type of atrial fibrillation, hypertension, diabetes mellitus, chronic kidney disease, cancer, stroke, transient ischemic attack, heart failure, coronary artery disease, major bleeding, aspirin use, statin use, P2Y12-inhibitor use and type of anticoagulation. Model 3 was additionally adjusted for high-sensitivity C-reactive protein. ALA = alpha-linolenic acid, CI = confidence interval, DHA = docosahexaenoic acid, DPA = docosapentaenoic acid, EPA = eicosapentaenoic acid.

Association of Omega-3 Fatty Acids with D-Dimer						
Omega-3 Fatty Acid	Model 1, *n* = 1096		Model 2, *n* = 1090		Model 3, *n* = 1088	
	Coefficient (Cl)	*p*-value	Coefficient (Cl)	*p*-value	Coefficient (Cl)	*p*-value
Total Omega-3 fatty acids	0.93 (0.89–0.97)	<0.001	0.93 (0.89–0.97)	0.001	0.94 (0.90–0.98)	0.004
Eicosapentaenoic acid (EPA)	0.88 (0.73–1.06)	0.185	0.92 (0.76–1.11)	0.398	0.91 (0.76–1.10)	0.336
Docosahexaenoic acid (DHA)	0.94 (0.88–1.01)	0.099	0.94 (0.88–1.00)	0.060	0.95 (0.89–1.01)	0.124
Docosapentaenoic acid (DPA)	0.95 (0.80–1.14)	0.591	0.92 (0.77–1.10)	0.379	0.93 (0.78–1.12)	0.452
Alpha-linolenic acid(ALA)	0.88 (0.58–1.34)	0.552	0.87 (0.57–1.32)	0.508	0.95 (0.63–1.45)	0.827

**Table 5:** Associations of *n*-3 FAs with beta-thromboglobulin. Model 1 was adjusted for age and sex. Model 2 was additionally adjusted for body mass index, physical activity, smoking, alcohol units per day, type of atrial fibrillation, hypertension, diabetes mellitus, chronic kidney disease, cancer, stroke, transient ischemic attack, heart failure, coronary artery disease, major bleeding, aspirin use, statin use, P2Y12-inhibitor use and type of anticoagulation. Model 3 was additionally adjusted for high-sensitivity C-reactive protein. ALA = alpha-linolenic acid, BTG = beta-thromboglobulin, CI = confidence interval, DHA = docosahexaenoic acid, DPA = docosapentaenoic acid, EPA = eicosapentaenoic acid.

Association of Omega-3 Fatty Acids with Beta-thromboglobulin (BTG)						
Omega-3 fatty acid	Model 1, *n* = 2371		Model 2, *n* = 2365		Model 3, *n* = 2339	
	Coefficient (Cl)	*p*-value	Coefficient (Cl)	*p*-value	Coefficient (Cl)	*p*-value
Total Omega-3 fatty acids	0.97 (0.96–0.99)	0.004	0.97 (0.95–0.99)	0.001	0.97 (0.95–0.99)	0.003
Eicosapentaenoic acid (EPA)	1.11 (1.02–1.20)	0.015	1.12 (1.03–1.22)	0.007	1.12 (1.03–1.22)	0.010
Docosahexaenoic acid (DHA)	0.95 (0.92–0.97)	<0.001	0.94 (0.91–0.97)	<0.001	0.94 (0.92–0.97)	<0.001
Docosapentaenoic acid (DPA)	0.93 (0.86–1.00)	0.053	0.91 (0.84–0.98)	0.018	0.91 (0.84–0.98)	0.019
Alpha-linolenic acid (ALA)	0.86 (0.71–1.03)	0.100	0.83 (0.69–1.00)	0.055	0.83 (0.69–1.01)	0.060

## Data Availability

Data will be made available on reasonable request.

## Abbreviations

AF	atrial fibrillation
ALA	alpha-linolenic acid
BTG	beta-thromboglobulin
DHA	docosahexaenoic acid
DPA	docosapentaenoic acid
EPA	eicosapentaenoic acid
IQR	interquartile range
*n*-3 FAs	Omega-3 fatty acids
SD	standard deviation
Swiss AF	Swiss atrial fibrillation
